# Production and Initial Characterization of Dad1p, a Component of the Dam1-DASH Kinetochore Complex

**DOI:** 10.1371/journal.pone.0003888

**Published:** 2008-12-09

**Authors:** Jennifer Waldo, Michael Scherrer

**Affiliations:** Department of Biology, SUNY New Paltz, New Paltz, New York, United States of America; National Cancer Institute, United States of America

## Abstract

In all dividing eukaryotic cells, the mitotic spindle (composed primarily of microtubules) must interact with chromosomes through a complex protein assembly called the kinetochore. In *Saccharomyces cerevisiae*, the Dam1-DASH complex plays an important role in promoting attachment between the kinetochore and the mitotic spindle. It also actively participates in the physical separation of sister chromatids in anaphase. Understanding the biochemical mechanisms used by Dam1-DASH has been facilitated by bacterial co-expression of the ten Dam1-DASH genes, which results in the production of a heterodecameric protein complex that can be studied *in vitro*. However, individual protein subunits are not soluble when expressed in *E. coli*, thus precluding analysis of the nature of the interaction between subunits and an examination of the assembly of the functional complex. In this paper, we describe the expression, solubilization, purification and refolding of Dad1p, one of the Dam1-DASH complex subunits. In addition, we show that Dad1p, when isolated in this manner forms dimers and/or tetramers, dependent upon protein concentration. This work provides an important tool for studying the Dam1-DASH complex that was previously unavailable, and provides an avenue of investigation for understanding how the individual heterodecamers associate with each other to facilitate chromosome segregation.

## Introduction

Cell division, in general, and chromosome segregation, in particular, are areas of immense concern to both the cell and the cell biologist. Understanding how these processes have evolved into reliable cellular functions is a goal of basic research and of interest to those pursuing drug development and design. In this field, one of the key areas of focus is the kinetochore. The kinetochore is the location where chromosomes interact with the mitotic spindle *via* an intricate dance that results first in the attachment of the sister chromatids to opposite spindle poles, and then in the active separation of sister chromatids from each other. For a limited number of species, the proteins that participate in mitosis by localizing to and functioning at the kinetochore have been catalogued. Of these, the best studied is the yeast *Saccharomyces cerevisiae* [1]. In this organism, there are believed to be at least 65 different proteins that work together to allow the kinetochore to function properly. An important goal in furthering our understanding of mitosis is to understand the contribution and regulation of each of these proteins.

Genetic and biochemical analyses have shown that many of the kinetochore proteins work within distinct protein assemblies, each with a particular localization and role in the process. One of the *S. cerevisiae* kinetochore components is the Dam1-DASH complex, which is composed of ten different protein subunits (Dam1p, Duo1p, Ask1p, Spc34p, Spc19p, Dadp1, Dad2p, Dad3p, Dad4p, Hsk3p) [2]–[5]. Dam1-DASH is an outer kinetochore protein complex, and is believed to interact directly with the microtubules. In fact, it can actually form a ring that encircles microtubules *in vitro*. It has been suggested that the ability of the Dam1-DASH complex to encircle microtubules is critical to its function in facilitating chromosome segregation in mitosis [6], [7], [8]. However, recent data supports the idea that the formation of intact Dam1-DASH rings around microtubules may not be required for its function [9], [10].

While different groups are investigating the role of ring formation by the Dam1-DASH complex, it is notable that even though the Dam1-DASH genes are essential in *S. cerevisiae*, homologues of these genes have not been identified in organisms other than the yeasts [2]. And, while the ten Dam1-DASH genes are essential in *S. cerevisiae*, they are not essential in *Schizosaccharomyces pombe*. If the functionality provided by Dam1-DASH in *S. cerevisiae* is evolutionary conserved outside of this species, it is possible that functional homologues exist in other cells that cannot be identified through sequence similarity. In order to support this hypothesis, a detailed understanding of how the Dam1-DASH complex functions is necessary to guide our search for the counterparts in other cells.

Biochemical and biophysical studies of the Dam1-DASH complex have been aided by the development of multi-cistronic expression vectors that allow purification of the intact complex from bacterial cells [7]. In addition, subassemblies of the Dam1-DASH complex can be co-expressed in and purified from *E. coli*. Such an approach has been utilized to show that the interaction of Dam1-DASH rings with microtubules is mediated primarily by the Dam1p and/or Duo1p subunits [11].

However, none of the individual Dam1-DASH subunits have yet been over-expressed in and purified from *E. coli*. This technical limitation restricts the types of experiments that can be conducted. For example, without access to individual subunits, understanding complex assembly is a difficult undertaking. In this paper, we report on the initial phases of our work to overcome this limitation. We have developed a strategy for efficient production of isolated Dad1p, one of the ten Dam1-DASH complex proteins. In an effort to better understand the contribution of this protein to Dam1-DASH kinetochore function, we show that isolated Dad1p is likely a tetramer or dimer. Thus, Dad1p may play a role in oligomerization of the Dam1-DASH ring, or other higher-order structures required for function at the kinetochore.

## Results

### Dad1p expression in *E. coli*

**Figure 1:**
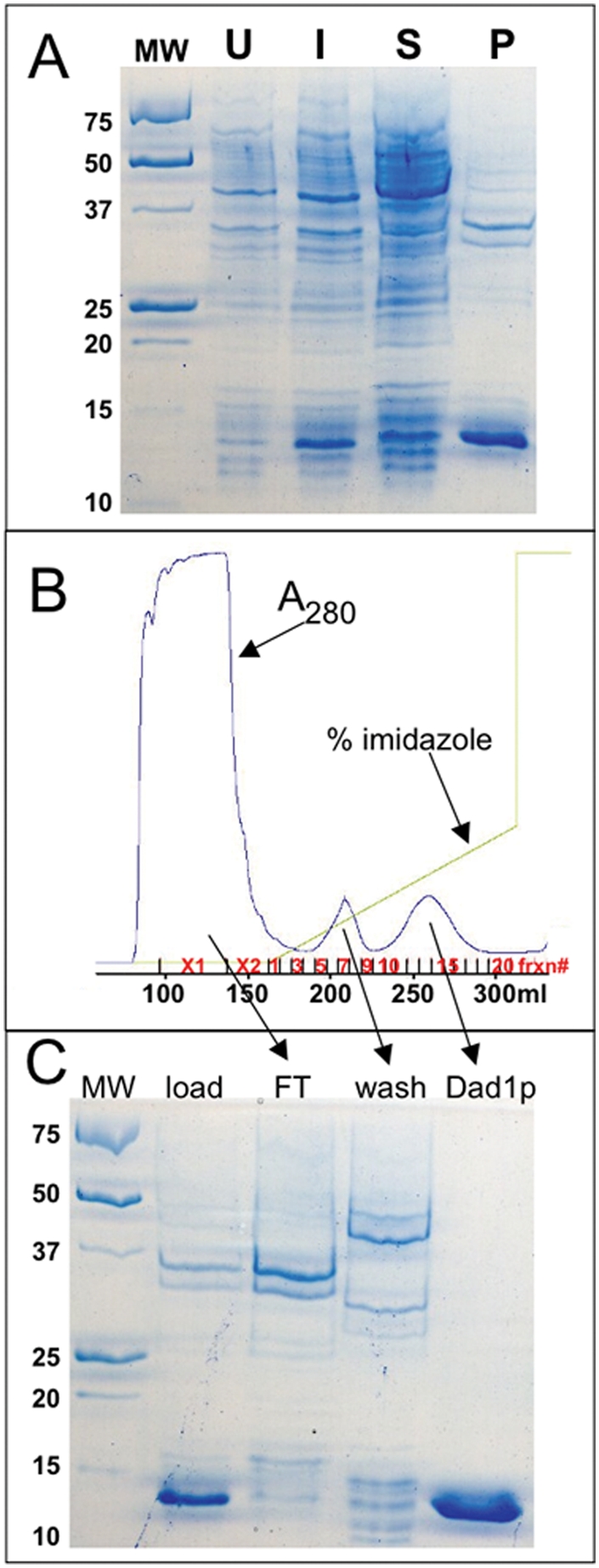
Expression and purification of Dad1p. A) Lane U contains a sample of uninduced cells, lane I contains a sample of IPTG-induced cells. Lane S contains soluble proteins and lane P contains insoluble proteins found in the pellet after centrifugation of the cell lysate. Sizes of molecular weight markers (in kDa) are indicated to the *left* of the gel. B) Cells were induced with IPTG to express Dad1p. The insoluble fraction was resolubilized in 8 M urea, clarified by centrifugation and loaded onto a Ni-sepharose column. The chromatograph from the Ni-sepharose column is shown. The *blue line* traces the elution of proteins as measured by A280. The *green line* traces increasing concentration of imidazole, which elutes bound proteins. Both elution volume (in ml) and fraction number (frxn#) are reported along the X-axis. C) SDS-PAGE analysis of selected fractions. Lanes containing the load onto the column, the flow-through (FT) (fraction X1), the wash (pooled fractions 5–9) and purified Dad1p (pooled fractions 12–16) are indicated *above* the gel.

We cloned the DAD1 gene into pET15b. This plasmid, when transformed into *E. coli* BL21*(DE3)*, produced Dad1p containing an N-terminal His_6_ tag. The over-expressed tagged protein migrates on SDS-PAGE at a position consistent with its calculated molecular weight of 13,053 Da (Figure 1A).

When these cells are harvested, lysed and clarified by centrifugation, the majority of the proteins are found in the soluble fraction, but Dad1p remains in the pellet (Figure 1A). To confirm that there is no soluble Dad1p, two approaches were taken. First, the soluble fraction was loaded onto a Ni-sepharose column, and subsequently eluted with a gradient of imidazole. No protein the size of Dad1p was observed in the eluate (data not shown). Second, the DAD1 gene was cloned into pET160, which produces a protein with a Lumio tag. This allows the expressed protein to be identified by adding a fluorescent reagent that specifically interacts with the tagged protein. Upon lysis, no fluorescent protein was observed in the soluble fraction (data not shown). The conclusion that Dad1p is not expressed in *E. coli* as a soluble protein is consistent with results published elsewhere [7].

We then took the pellet fraction and solubilized it by the addition of 8 M urea. The sample was then loaded onto a Ni-sepharose column in the presence of 8 M urea and eluted with a gradient of imidazole (Figure 1B). This results in a preparation of Dad1p that is >95% pure, as judged by Coomassie-stained SDS-PAGE gels (Figure 1C).

### Dad1p refolding

**Figure 2:**
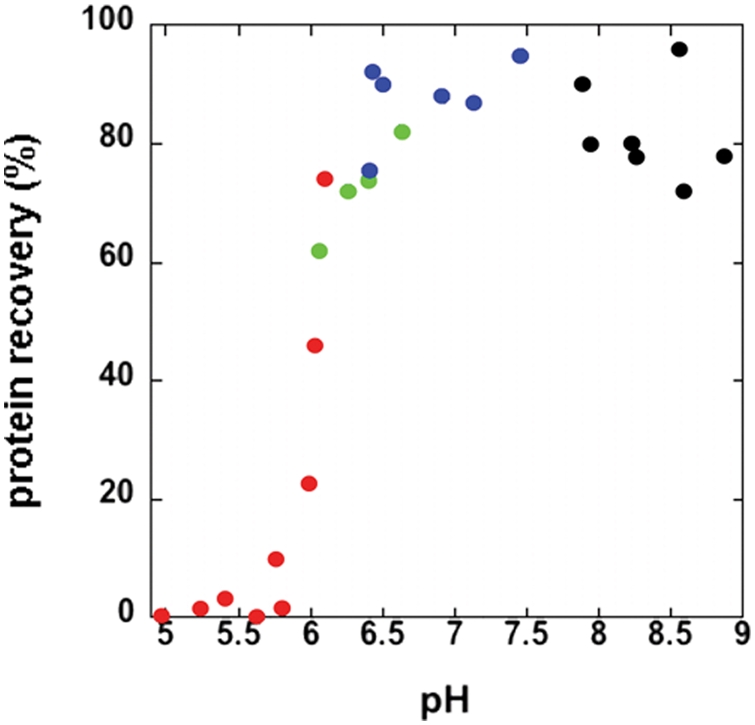
Re-solubilization of Dad1p is pH dependent. The pool of purified Dad1p was dialyzed against buffers at different pH and then centrifuged to separate the soluble and insoluble components. Protein concentration of the soluble fraction was determined and the results are reported as % recovery (the amount of protein present following dialysis divided by the total amount of protein in each sample). Samples were dialyzed against solutions containing Sodium Acetate (red), PIPES (green), HEPES (blue) or Tris (black), 100 mM NaCl and 2 mM EDTA.

The goal of our study was to produce Dad1p that had folded into its native conformation. To facilitate the re-folding of the protein upon removal of the urea, we took a sample of the purified Dad1p and dialyzed it against a buffered solution without urea. This experiment was repeated at a number of different pH values. Following overnight dialysis, samples were centrifuged to remove any unfolded, precipitated protein. These results are presented in Figure 2.

There is a strict pH-dependence to re-folding of Dad1p. At pH below ∼6, the protein is virtually 100% precipitated, while at higher pH values, the vast majority of the protein is soluble. The results shown in Figure 2 include experiments conducted with 100 mM NaCl present. Because salt concentration can affect a protein's stability, we repeated the re-folding at 0, 0.5 and 1 M NaCl. In all of these cases, there was no affect on the pH-dependence of re-folding observed at 100 mM NaCl (data not shown).

### Dad1p characterization

While the Dad1p obtained following dialysis was soluble, we were very interested in assessing the quality of the protein. To address this issue, we performed two chromatographic techniques on the purified and re-folded Dad1p: high-resolution ion exchange chromatography and gel filtration.

Applying the re-folded Dad1p to a MonoQ column results in a single peak of protein upon elution with increasing amount of salt (data not shown). This suggests that the re-folded Dad1p is behaving as a single species, rather than as a mixture of alternate assemblies with different electrostatic characteristics.

**Figure 3:**
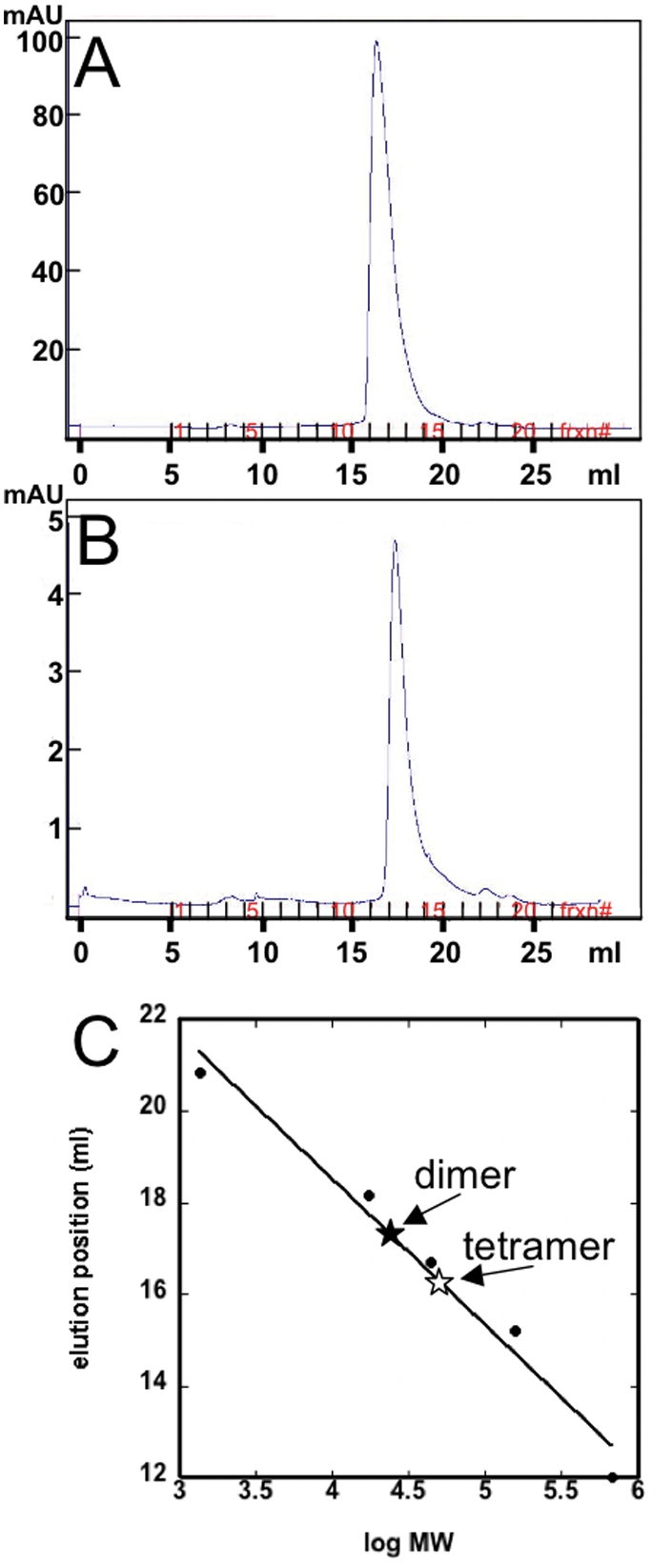
Characterization of Dad1p. A) When applied to a Superdex 200 gel filtration column, the re-solubilized Dad1p (∼10 mg/ml) elutes in a single peak. B) When applied to a Superdex 200 gel filtration column, a more dilute sample of Dad1p (∼1 mg/ml) elutes slightly later. C) A comparison of the elution position of 10 mg/ml (*open star*) and 1 mg/ml (*filled star*) Dad1p. The elution positions for the following standards are included: thyroglobulin (670 kDa), γ-globulin (158 kDa), ovalubumin (44 kDa), myoglobin (17 kDa) and vitamin B_12_ (1,350 Da).

To assess the size of the re-folded Dad1p, we applied a sample to a Superdex 200 gel filtration column. This results in the elution of protein in a single peak (Figure 3A), suggesting that the re-folded Dad1p is uniform in its oligomeric state and does not contain any detectable large-size protein aggregates.

Interestingly, Dad1p exhibits concentration-dependent oligomerization. At high concentrations (∼10 mg/ml), Dad1p elutes from the column slightly earlier (Figure 3A) than when the protein concentration is lower, at approximately 1 mg/ml (Figure 3B). When the gel filtration elution position is compared to proteins of known size applied to the same column under the same conditions (Figure 3C), the apparent molecular weight of Dad1p can be ascertained. The peak produced with the higher concentration of Dad1p is calculated to represent a molecular weight of 48,305 Da. Since an individual Dad1p is 13,053Da, this suggests ∼4 Dad1p are present per complex. At the lower protein concentration, the apparent molecular weight is 24,963 Da, suggesting ∼2 Dad1p per complex.

## Discussion

Many of the protein players at the kinetochore appear to be conserved across eukaryotic species. Interestingly, this is not true for the Dam1-DASH complex, as no Dam1-DASH complex homologues are identifiable outside of the yeasts. In addition, all yeasts appear to have Dam1-DASH complex, regardless of whether they use a specific sequence to identify the location of the kinetochore, as in *S. cerevisiae*, or if they have a regional kinetochore assembly process, as in *S. pombe* [12]. Because Dam1-DASH is involved in the both types of centromeres, it is possible that the function it provides for cells is universal in nature, rather than being yeast-specific.

In an effort to better understand the biochemistry of the Dam1-DASH complex, we have set out to establish protocols for the purification of individual Dam1-DASH complex subunits. This effort is hampered by the fact that the individual subunits are not soluble when expressed in *E. coli*. However, we show here that Dad1p can be purified from inclusion bodies and re-folded into a soluble, non-aggregated conformation.

Our studies also suggest that Dad1p likely exists in a dimer-tetramer equilibrium *in vitro*, and that oligomerization state is influenced by concentration. What does this suggest about how Dad1p may function in cells? In the cell, Dad1p is associated with the nine other proteins that comprise the Dam1-DASH complex. We do not yet know if it functions independently of this complex. If rings around microtubules are required in mitosis, ∼650 Dad1p would be needed [7], [14]. Under this scenario, free Dad1p would likely not be found, as the reported abundance of Dad1p in cells is ∼799 molecules [13]. Therefore, isolated oligomers of Dad1p may not be significant players in the cell. Instead, the ability of Dad1p to oligomerize may be required for building rings by facilitating interactions between adjacent Dam1-DASH complexes. A role for oligomerization could be postulated even if ring formation is not required for Dam1-DASH complex function. In experiments suggesting that ring formation may not be necessary, multimers of Dam1-DASH were still observed and were important for chromosome segregation [9], [10].

In *S. cerevisiae*, the Dam1-DASH complex associates with the kinetochore throughout the cell cycle [15]. However, in *S. pombe*, the Dam1-DASH complex is found at kinetochores only during mitosis [16], [17], [18]. One exception to this is the observation of Dad1 at *S. pombe* kinetochores in interphase cells [16], [17], [18]. Therefore, future research may indeed reveal a role for Dad1p that is independent of its Dam1-DASH complex binding partners. The ability to produce Dad1p as an isolated protein will enhance our ability to examine this possibility.

## Materials and Methods

### Cloning *DAD1*

Genomic DNA from *S. cerevisiae* was extracted by lysing cells in the presence of Instagene Matrix (BioRad). Two primers were designed and ordered from Operon to facilitate amplification of the gene by PCR (F1: 5′ ACTCGAGATGATGGCTAGTACATCC3′ and R1: 5′AGGATCCTTACTTCGTTTTCGATTGAGA3′). Both the purified PCR product and pET15b plasmid (Novagen) were incubated with BamHI and XbaI (NEB), purified via spin columns (Qiagen), ligated (NEB) together and transformed into Top10 cells (Invitrogen). Correct plasmid construction was verified by PCR, restriction enzyme analysis and DNA sequencing. pET15b-*DAD1* was transformed into competent BL21*(DE3)* cells for protein expression studies.

### Dad1p expression, urea solubilization and initial purification

BL21*(DE3)* cells harboring pET15-*DAD1* were inoculated into LB plus 100 µg/ml ampicillin and grown to saturation overnight. These cultures were then used to inoculate 1L cultures the following morning. The 1L cultures were grown to OD = 0.4 at 37°C and protein expression was induced by the addition of 0.5 mM IPTG. Cells were harvested by centrifugation at 4°C after four hours growth and frozen at −80°C.

Cells were thawed in buffer A (50 mM Tris pH 8.0, .5 M NaCl) supplemented with lysozyme and sonicated. After centrifugation at 12,000×g for 30 minutes at 4°C, the pellet was resuspended in buffer A containing 8 M urea. The solution was centrifuged at 12,000×g for 30 minutes at 4°C. The supernatant was loaded onto a 10 ml column of chelating sepharose charged with nickel sulfate (GE Healthcare). The column was washed with Buffer A containing 8 M urea. Proteins were eluted by applying a linear gradient of immidazole (0 to .5 M) in the presence of 8 M urea. Fractions containing Dad1p were identified by SDS-PAGE. All chromatography steps were performed on an AKTA-FPLC (GE Healthcare).

### Re-folding by dialysis

One ml samples of the pooled fractions from the Nickel-sepharose column were placed into dialysis tubing and equilibrated overnight at 4°C against 1L of solutions buffered at various pHs. All solutions contained .1 M NaCl, and 50 mM buffer. For the pH range of 5 to 6, Sodium Acetate served as the buffer. For the pH range of 6 to 6.8, Pipes served as the buffer. For the pH range 6.5 to 7.5, Hepes was the buffer. For the pH range 7.5 to 9, Tris was the buffer. The pH of the buffer was measured after dialysis. Protein concentration was measured by the Bradford method (BioRad) before and after dialysis, following a 15-minute 13 K centrifugation step at 4°C. Additional purification steps: Following overnight dialysis into Buffer B (20 mM Tris, pH 7.5, 0.1 M NaCl, 2 mM EDTA), the sample was centrifuged and bound to a 1 ml monoQ column. The column was eluted with 20 mM Tris, pH 7.5, 0.6 M NaCl, 2 mM EDTA. Fractions containing Dad1p were pooled and concentrated with an Amicon Ultra 10.

Gel filtration analysis was conducted with a Superdex 200 (GE Healthcare) column equilibrated in buffer B. The column was calibrated with an equal volume of gel filtration standards (BioRad).
